# Combination Foretinib and Anti-PD-1 Antibody Immunotherapy for Colorectal Carcinoma

**DOI:** 10.3389/fcell.2021.689727

**Published:** 2021-07-08

**Authors:** Yuyin Fu, Yujia Peng, Shengyan Zhao, Jun Mou, Lishi Zeng, Xiaohua Jiang, Chengli Yang, Cheng Huang, Yuyan Li, Yin Lu, Mengdan Wu, Yanfang Yang, Ting Kong, Qinhuai Lai, Yangping Wu, Yuqin Yao, Yuxi Wang, Lantu Gou, Jinliang Yang

**Affiliations:** ^1^State Key Laboratory of Biotherapy and Cancer Center/Collaborative Innovation Center for Biotherapy, West China Hospital, Sichuan University, Chengdu, China; ^2^Laboratory of Infectious Diseases and Vaccine, West China Hospital, Sichuan University, Chengdu, China; ^3^Department of Clinical Research Management, West China Hospital, Sichuan University, Chengdu, China; ^4^West China School of Public Health and Healthy Food Evaluation Research Center/No. 4 West China Teaching Hospital, Sichuan University, Chengdu, China; ^5^Department of Respiratory and Critical Care Medicine, West China Hospital, Sichuan University, Chengdu, China

**Keywords:** foretinib, anti-PD-1, combination therapy, immunotherapy, tumor microenvironment, colon cancer

## Abstract

Immune checkpoint inhibitors have achieved unprecedented success in cancer immunotherapy. However, the overall response rate to immune checkpoint inhibitor therapy for many cancers is only between 20 and 40%, and even less for colorectal cancer (CRC) patients. Thus, there is an urgent need to develop an efficient immunotherapeutic strategy for CRC. Here, we developed a novel CRC combination therapy consisting of a multiple receptor tyrosine kinase inhibitor (Foretinib) and anti-PD-1 antibody. The combination therapy significantly inhibited tumor growth in mice, led to improved tumor regression without relapse (83% for CT26 tumors and 50% for MC38 tumors) and prolonged overall survival. Mechanistically, Foretinib caused increased levels of PD-L1 via activating the JAK2-STAT1 pathway, which could improve the effectiveness of the immune checkpoint inhibitor. Moreover, the combination therapy remodeled the tumor microenvironment and enhanced anti-tumor immunity by further increasing the infiltration and improving the function of T cells, decreasing the percentage of tumor-associated macrophages (TAMs) and inhibiting their polarization toward the M2 phenotype. Furthermore, the combination therapy inhibited the metastasis of CT26-Luc tumors to the lung in BALB/c mouse by reducing proportions of regulatory T-cells, TAMs and M2 phenotype TAMs in their lungs. This study suggests that a novel combination therapy utilizing both Foretinib and anti-PD-1 antibody could be an effective combination strategy for CRC immunotherapy.

## Introduction

Colorectal cancer (CRC) is the third most commonly diagnosed cancer and the second leading cause of cancer-related deaths worldwide. Over 1.8 million new CRC cases and 881,000 deaths were estimated to occur in 2018 (Bray et al., 2018). Whilst there are many advanced diagnostic and therapeutic methods for CRC, it remains one of the major cancers with a relatively high mortality rate.

Immune checkpoint inhibitors (ICIs) that target PDCD1/CD274(PD-1/PD-L1) have been used to treat a variety of malignant tumors, including melanoma, lung cancer, renal cell cancer, head and neck cancer, Hodgkin’s disease, urothelial cancer (Ribas and Wolchok, 2018; Xiao et al., 2020) and CRC with deficient mismatch repair (dMMR) or microsatellite instability-high (MSI-H). But CRC with dMMR/MSH-H only accounts for approximately 15% of all advanced CRC cases, therefore the majority of patients cannot benefit ICIs (Overman et al., 2017; Oliveira et al., 2019). The poor therapeutic effect for CRC patients is associated with the immunosuppressive tumor microenvironment (TME), which causes resistance to immune checkpoint blockade (ICB) (Pitt et al., 2016). Hence, there is an urgent need to overcome the CRC associated immunosuppression, which would enable the proportion of patients who benefit from PD-1/PD-L1 blockade to be expanded. Numerous preclinical studies have indicated that the combination of ICIs with other treatments such as chemotherapy, radiation therapy and targeted therapy could be a promising approach to overcome immunosuppression and improve therapeutic efficacy (O’Neill and Cao, 2019).

Low T cell infiltration, especially CD8^+^ T cells or a high proportion of tumor-associated macrophages (TAMs) appear to be associated with reduced antitumor drug efficacy and seem to be associated with poor clinical outcomes in most carcinoma cases (Huang et al., 2017; Xu et al., 2018; Feng et al., 2019). Indeed, CD8^+^ T cells are considered major drivers of antitumor immunity in tumors. These cells can specifically recognize and kill cancer cells via the release of cytotoxic molecules and cytokines (van der Leun et al., 2020). But, as the major tumor-infiltrating immune cell population, TAMs are commonly hijacked by tumor cells to inhibit the function of T cells and promote an immunosuppressive TME, ultimately leading to tumor growth, immunoevasion, metastasis and angiogenesis (De Palma and Lewis, 2013; Chen et al., 2018; Jeong et al., 2019). Previous studies have indicated that increased T cell or decreased TAM infiltration in tumors is correlated with a favorable prognosis and has a synergetic effect with anti-PD-1 immunotherapy.

Foretinib is an available inhibitor of multiple receptor tyrosine kinases (RTKs), including vascular endothelial growth factor receptor 2 (VEGFR2) and c-MET. It can be taken orally and has been demonstrated to have significant activity against a wide range of tumors *in vitro* and *in vivo* (Huynh et al., 2012; Choueiri et al., 2013; Faria et al., 2015; Yau et al., 2017). However, to date the impact of Foretinib on antitumor immunity has not been clarified. This study demonstrates that Foretinib increased PD-L1 expression via the activation of the JAK2-STAT1 pathway and improved the immune microenvironment by increased the infiltration of T cells. This provided the rationale for combining PD-1/PD-L1 inhibitors with Foretinib. The combination of Foretinib with anti-PD-1 antibody significantly inhibited the growth of MC38 and CT26 tumors by further enhancing the infiltration and function of T cells, decreasing the proportion of TAMs and inhibiting their polarization toward the M2 phenotype. Furthermore, the combination therapy significantly inhibited the CT26-Luc tumor metastasis to the lung in BALB/c mouse by reducing proportions of regulatory T cells (Tregs), TAMs and M2 phenotype TAMs in the lung. The results of this investigation have enabled proposition of a novel combination strategy to enhance the effects of immunotherapy for CRC.

## Results

### Foretinib Increased the Expression of PD-L1 by Activating the JAK2-STAT1 Pathway

**FIGURE 1:**
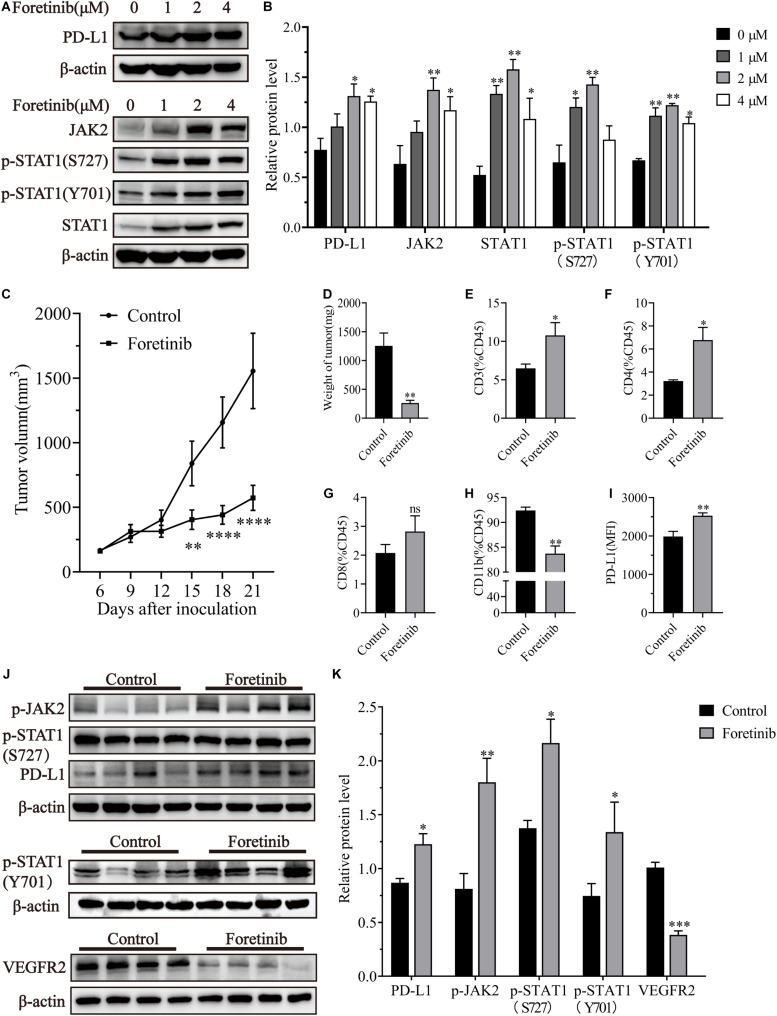
Foretinib increased PD-L1 and enhanced the infiltration of T cells in MC38 tumors. The expression of PD-L1, JAK2, STAT1, phospho-STAT1(S727), phospho-STAT1(Y701) and β-actin was detected by Western-blotting in MC38 colon cancer cells, which were treated with different concentrations of Foretinib for 24 h. The relative protein levels were shown in figures **(A,B)**. MC38 cells (1 × 10^6^) were transplanted subcutaneously into the right flank of C57BL/6 mice. Six days after transplantation (a tumor volume nearly of 100 mm^3^), the mice were randomly allocated to either the control or treatment groups. Drugs were administered as described in the “Materials and Methods” section and the tumor volumes were measured every 3 days **(C)**, six mice were allocated per group (*n* = 6). After the treatment was completed, the tumors were harvested and weighed **(D)** and the infiltration of CD3^+^ **(E)**, CD4^+^ **(F)**, CD8^+^ **(G)**, and CD11b^+^ **(H)** cells was determined and the mean fluorescence intensity (MFI) of PD-L1 **(I)** in the tumors was determined by FCM. The relative protein levels in the tumor tissue for phospho-JAK2, STAT1, phospho-STAT1(S727), phospho-STAT1(Y701), PD-L1, VEGFR2, and β-actin were determined using Western-blotting **(J,K)** (*n* = 4 mice). The data was presented using the mean ± SEM where applicable, * (*P* ≤ 0.05), ** (*P* ≤ 0.01), *** (*P* < 0.001), **** (*P* < 0.0001) and ns (no statistical significance, *P* ≥ 0.05).

To investigate the effect of Foretinib on PD-L1 expression in colon cancer cells, different concentrations of Foretinib (0, 1, 2, 4 μM) were used to treat MC38 murine tumor cells for 24 h, after which the protein levels were analyzed by western blotting. These results demonstrated that the levels of PD-L1, JAK2, STAT1, phospho-STAT1(S727), phospho-STAT1(Y701) were elevated following Foretinib treatment (Figures 1A,B). The same changes were also observed in CT26 murine colon cancer cells and in HCT116, HT29 and SW480 human colon cancer cells (Supplementary Figure 1). Moreover, the expression of PD-L1, JAK2 phosphorylation and STAT1 phosphorylation (included S727 and Y701) were increased in MC38 tumor tissues from C57BL/6 mice after Foretinib treatment (Figures 1J,K).

### Foretinib Enhanced T Cell Infiltration *in vivo*

Foretinib significantly inhibited MC38 tumor growth *in vivo* compared to the control (Figures 1C,D). To determine the immunomodulatory functions of Foretinib, the MC38 subcutaneous model was used. The infiltration of T cells, including CD4^+^ and CD8^+^ T cells (Figures 1E–G), were enhanced; whereas, the CD11b^+^ cells (Figure 1H), which comprise abundant immunosuppressive cells such as TAMs and Myeloid-derived suppressor cells (MDSCs), were decreased in the tumors after Foretinib treatment. The PD-L1 expression on tumor cells (CD45^–^) was also enhanced after Foretinib treatment (Figure 1I). Additionally, the number of T cells in the peripheral blood and spleen were increased after Foretinib treatment (Supplementary Figure 2). These results demonstrated that Foretinib could regulate the immune cells associated with colon cancer *in vivo*. It has previously been reported that inhibited the expression of VEGFR2 could ameliorate the TME and improve immunotherapy outcomes (Fukurnura et al., 2018; Khan and Kerbel, 2018). Consistent with previous reports, Foretinib significantly inhibited VEGFR2 expression in MC38 tumors (Figures 1J,K).

### Combined Treatment of Foretinib and αPD-1 Significantly Inhibited the Growth of MC38 Tumors and Prolonged the Survival Rate

**FIGURE 2:**
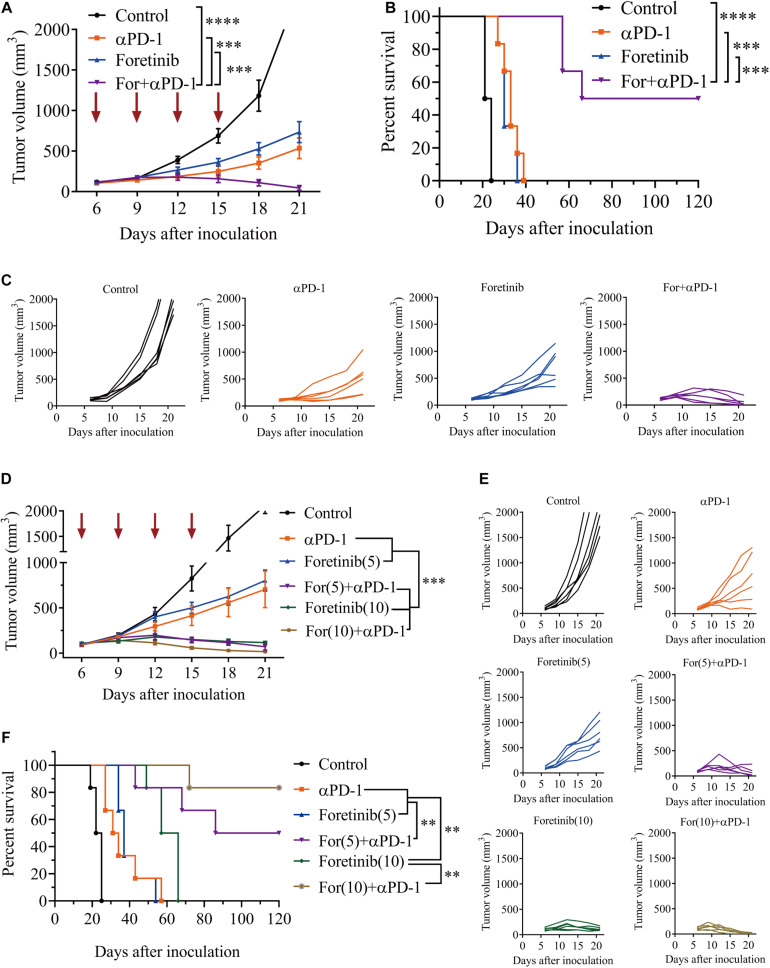
Foretinib synergistically enhanced the anti-tumor function of the αPD-1 antibody *in vivo*. MC38 cells subcutaneously implanted into the mice and when the volume reached approximately 100 mm^3^, the mice were randomly allocated to either the control or treatment groups (*n* = 6). The drugs were given as described in the “Materials and Methods” section. **(A)** The tumor volume was measured every 3 days. **(B)** The Kaplan–Meier survival distribution in the model is displayed for the mice following the treatment protocol. **(C)** Tumor growth curves for the mouse in each group are displayed. **(D–F)** The tumor volume and survival data were shown for mice with MC38 tumors that received the increased Foretinib dose (10 mg/kg). The data is presented using the mean ± SEM where applicable, * (*P* ≤ 0.05), ** (*P* ≤ 0.01), *** (*P* < 0.001), **** (*P* < 0.0001), and ns (no statistical significance, *P* ≥ 0.05).

The results described above prompted an investigation into the *in vivo* antitumor effects of Foretinib when combined with anti-PD-1 (αPD-1) therapy. Tumor growth was inhibited by Foretinib (67.97% inhibition) and αPD-1 (76.69% inhibition), moreover the inhibitory effect was significantly enhanced after the combination treatment (For+αPD-1, 98.05% inhibition) (Figures 2A,C) in the murine MC38 colon cancer subcutaneous model. This combination led to complete tumor regression in 50% (three out of six mice), for which a recurrence-free survival pattern over 120 days was observed (Figure 2B). With an increased dose of 10 mg/kg, the inhibitory effect of Foretinib was enhanced (Figures 2D,E), with slow tumor regrowth observed after the Foretinib treatment. There was an 83% complete rate of tumor regression without recurrence over 120 days after the combination treatment of the higher-dose (10 mg/kg) of Foretinib and αPD-1 (Figure 2F) in the murine MC38 colon cancer subcutaneous model. These results suggest that Foretinib has synergistic antitumor activity when combined with αPD-1, and that the synergistic antitumor efficacy was Foretinib dosage-dependent.

### Combined Treatment Enhanced T Cell Infiltration and Induces CD8^+^ T Cell-Dependent Anti-tumor Immune Response

**FIGURE 3:**
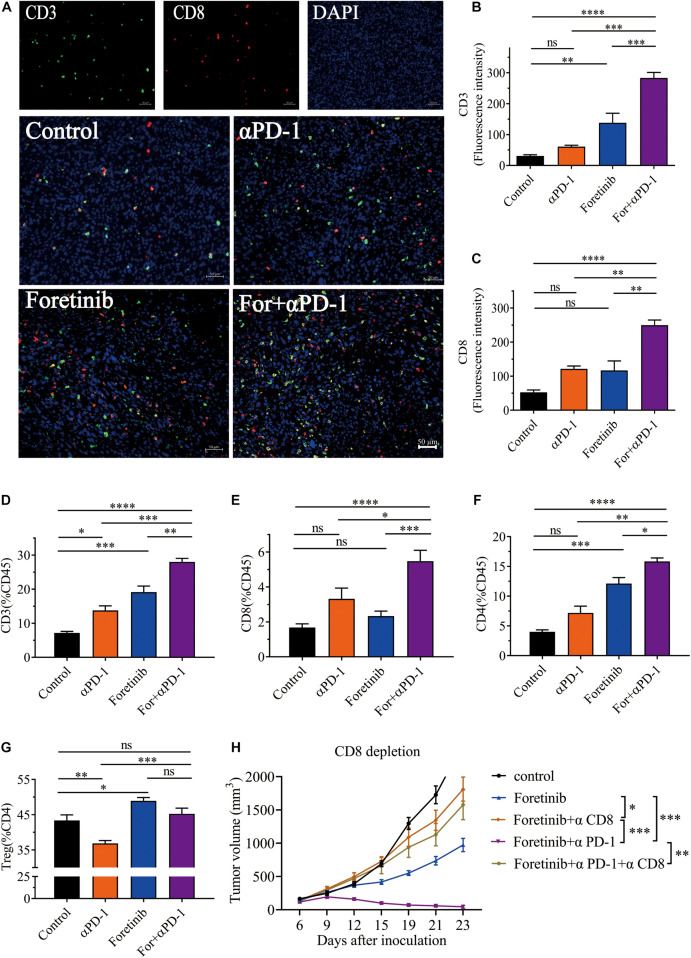
Combination therapy enhanced T cell infiltration and induces CD8^+^ T cell-dependent anti-tumor immune response. After the treatment was completed, T cell infiltration in MC38 tumor tissue was detected by immunohistochemistry. Representative immunofluorescent images of CD3, CD8, DAPI nuclear staining and the merge picture from each group **(A)**, and the fluorescence intensity of CD3 **(B)** and CD8 **(C)** were quantified in the tumor tissues. Scale bar, 50 μm (*n* = 5 mice per group). **(D–F)** Percentages of CD3^+^ T cells, CD8^+^ T cells and CD4^+^ T cells in tumor. **(G)** The percentages of Tregs (CD25^+^FoxP3^+^), gated on CD4^+^ T cells. **(H)** Tumor growth of CD8^+^ T cell depletion assay (*n* = 6 mice per group). The data is presented using the mean ± SEM where applicable, * (*P* ≤ 0.05), ** (*P* ≤ 0.01), *** (*P* < 0.001), **** (*P* < 0.0001), and ns (no statistical significance, *P* ≥ 0.05).

T cells play a key role in antitumor immunity, therefore the T cell infiltration in tumor tissues after combined treatment was determined via immunohistochemistry (IHC). The IHC results showed the tumor in the combination therapy contained more CD3^+^ and CD8^+^ T cells than the αPD-1 and Foretinib monotherapy or the control group (Figures 3A–C). To validate this result, we detected T cell in TME by flow cytometry (FCM). Consistent with IHC results, the combination therapy exhibited increased T cell infiltration (Figures 3D–F) in the TME when compared to the control and those treated with Foretinib or αPD-1 alone. However, no statistically significant changes were identified for Tregs in tumors following combination therapy compared to the control (Figure 3G). Additionally, these results demonstrated that αPD-1 therapy decreased the portion of Tregs, while Foretinib enhanced Tregs infiltration in the TME, which is similar to some other multi-target kinase inhibitors (Kwilas et al., 2014; Chen et al., 2015). The conflicting roles of Foretinib and αPD-1 may explain why there were no obvious changes to the Tregs following the combination therapy. These results revealed that the combination therapy significantly enhanced the T cell infiltration in the tumor.

To determine the functionality of T cells after treatment, the secretion of IFN-γ in T cells and the expression of PD-1 on T cells that was a co-inhibitory molecule and linked to T cell dysfunction, were evaluated by FCM. The results demonstrated that the IFN-γ^+^ cells in CD4^+^ T cells (Supplementary Figure 3A) and CD8^+^ T cells (Supplementary Figure 3B) were increased following each treatment, with combination therapy displaying the greatest change. And the PD-1^+^ T cells were decreased after treatment, especially after combination therapy (Supplementary Figures 3C–E). At the same time, we detected the IFN-γ and TNF-α in plasma after treatments and the result demonstrated that the combination therapy increased both cytokines concentration in peripheral blood (Supplementary Figures 3F,G). This result suggested that the combination therapy enhanced the function of T cells *in vivo*, which when combined with the aforementioned results, suggests that the combination of Foretinib and αPD-1 significantly enhanced the infiltration and function of T cells and promoted antitumor immunity.

In order to validate CD8^+^ T cells are responsible for tumor cell killing, the CD8^+^ T cells were depleted. The result showed that the MC38 tumor bearing mice with depleted CD8^+^ T cells showed complete abrogation of tumor rejection (Figure 3H). Hence, it is evident that the CD8^+^ T cells are required to achieve therapeutic efficacy.

In summary, these results indicated that the combination treatment enhanced the infiltration and function of T cells in tumor microenvironment and elicited a CD8^+^ T cell-dependent anti-tumor immune response.

### Combined Treatment Reversed TAM Mediated Immunosuppression and Inhibited Angiogenesis

**FIGURE 4:**
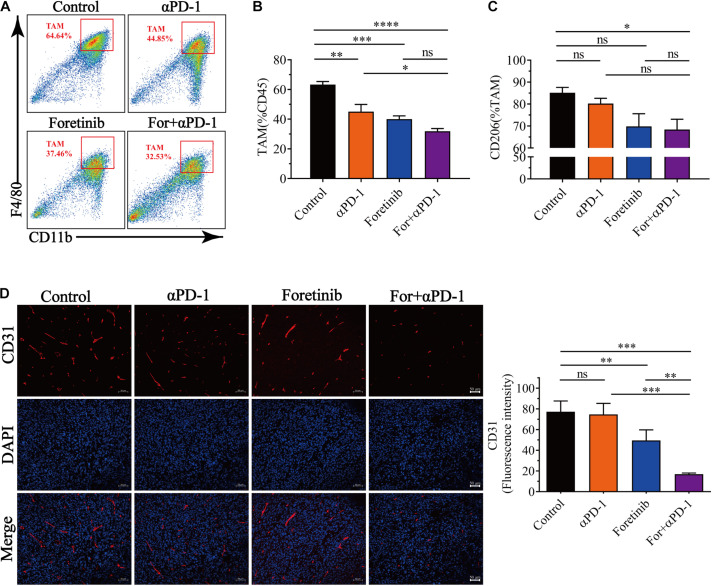
Combination therapy decreased the number of TAM cells and inhibited angiogenesis. After finishing the treatment protocol, the tumor tissues were harvested and the TAMs and M2-type TAM infiltration was detected by FCM (*n* = 6), angiogenesis (CD31+ cells) were detected by immunofluorescent (*n* = 5). **(A)** Representative images of the gating strategy to defined TAMs. TAMs were characterized as being CD11b^+^F4/80^+^. **(B)** Percentage of TAMs from each group. **(C)** Percentage of CD206 expression in TAMs from each group. **(D)** Representative immunofluorescent images for the CD31 (Red) and DAPI nuclear staining (Blue) from each group (left) and fluorescence intensity of CD31 (right) was quantified in the tumor tissues. Scale bar, 50 μm. The data is presented using the mean ± SEM where applicable, * (*P* ≤ 0.05), ** (*P* ≤ 0.01), *** (*P* < 0.001), **** (*P* < 0.0001), and ns (no statistical significance, *P* ≥ 0.05).

It has been widely reported that the infiltration of TAMs in tumors is associated with poor prognosis. To study the effects of the combined treatment on TAMs within the TME, the TAM population and functional tumor signaling molecules were assessed. 60% of the immune cells in the TME were observed to be TAMs for the control and all the treatments inhibited the recruitment of TAMs into the TME (Figures 4A,B). Moreover, the treatments inhibited TAM polarization toward the M2 phenotype and the proportion of CD206^+^ cells was reduced, especially following the combination treatment (Figure 4C). Taken together, the combination therapy not only reduced the number of TAMs but also inhibited TAMs polarization toward the M2 phenotype.

Multi-target kinase inhibitors such as Apatinib and Regorafenib for angiogenesis inhibition have not only been shown inhibit TAM recruitment into tumors, but also enhanced antitumor immune functions (Tsai et al., 2017; Zhao et al., 2019; Wang et al., 2020). IHC was used to evaluate the effects of the combination treatment on angiogenesis. In accordance with previous studies, Foretinib significantly inhibited angiogenesis in tumor tissues, as evidenced by reduced vascular density (CD31^+^) when compared to the αPD-1 treatment or control. The combination treatment yielded a further reduction in vascular density compared to Foretinib alone (Figure 4D). These results indicated that the combination therapy lessened the hypervascularization of the tumors and inhibited the recruitment of TAMs.

Additionally, the MDSC (CD11b^+^Gr-1^+^) population was assessed by FCM due to their immunosuppressive effects in tumors. However, the number of MDSCs in MC38 tumors were not significantly different despite a slight increase following treatment (Supplementary Figure 4A).

### Combined Treatment Abrogates the Tumor Development in the CT26 Colon Cancer Model

**FIGURE 5:**
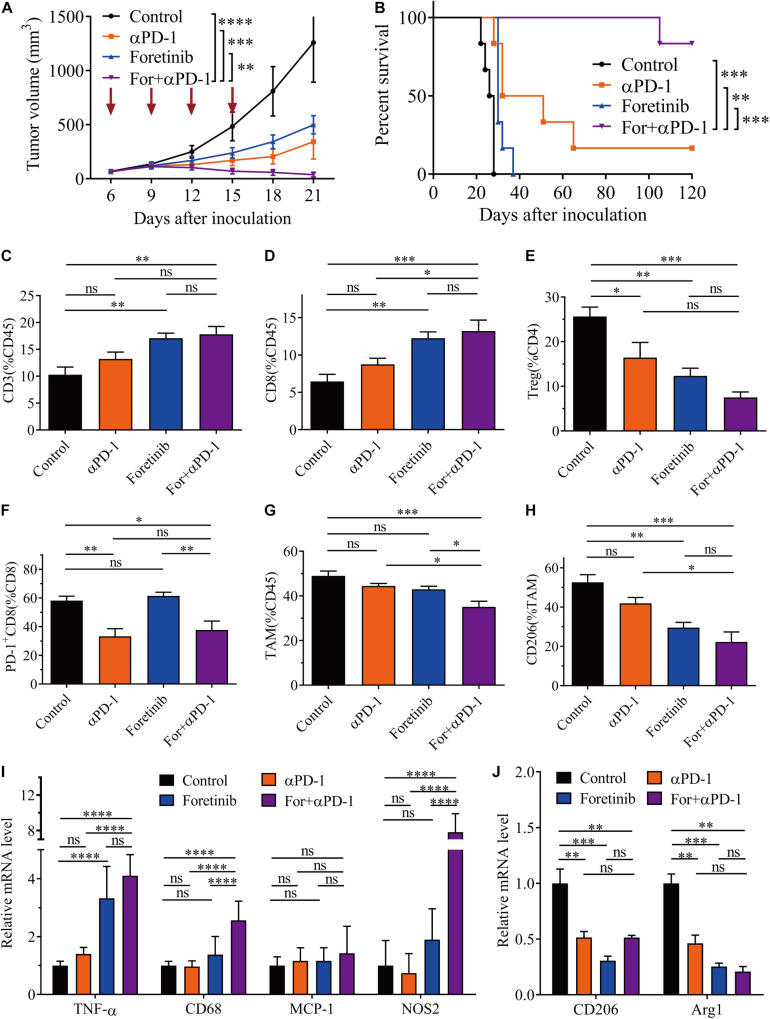
Combination therapy ameliorated the TME in CT26 tumors. CT26 cells (5 × 10^5^) were transplanted subcutaneously into the back of BALB/c mice. When the tumor volume reached 50–100 mm^3^ (6 days after transplantation), the mice were randomly allocated to either the control or treatment groups. The drugs were given as described in “Materials and Methods” section. **(A)** Tumor volumes were monitored every 3 days and **(B)** the Kaplan–Meier survival distribution was plotting for the mice following the treatment protocol (*n* = 6). After treatment, the infiltration of CD3^+^ T cells **(C)**, CD8^+^ T cells **(D)**, and Tregs **(E)**, plus the expression of co-inhibitory molecules (PD-1) on CD8^+^ T cells **(F)** was detected via FCM. The percentage of TAMs **(G)** and the expression of CD206 on TAMs **(H)** was detected by FCM (*n* = 5). **(I,J)** qRT-PCR analysis of TAM function-associated gene expression (*n* = 4). The data is presented using the mean ± SEM where applicable, * (*P* ≤ 0.05), ** (*P* ≤ 0.01), *** (*P* < 0.001), **** (*P* < 0.0001), and ns (no statistical significance, *P* ≥ 0.05).

To verify the effect of the combination treatment in another colon cancer allograft model, the combination strategies were employed in the murine CT26 subcutaneous colon cancer model. The combination therapy was demonstrated to be significantly more effective than either monotherapy and induced complete tumor clearance in 83% (five out of six mice) of the mice and prolonged overall survival (Figures 5A,B). An analysis of the immune cells in the TME following treatment demonstrated that the T cell population significantly increased, especially CD3^+^ and CD8^+^ T cells following combination treatment (Figures 5C,D). Moreover, the number of Tregs were significantly reduced following each treatment, particularly the combination treatment (Figure 5E). Interestingly, the Tregs in the CT26 tumor and MC38 tumor had opposite trends after Foretinib treatment (Figures 3F, 5E). Moreover, the genes associated with T cell function (e.g., perforin and IFN-γ) and T cell recruitment (e.g., CCL5) (Supplementary Figures 5A,B) were enriched within the tumor and the level of IFN-γ and TNF-α (Supplementary Figures 5C,D) in plasma after treatments was elevated, especially following the combined treatment.

The expression levels of PD-1 on CD3^+^ and CD8^+^ T cells were decreased following αPD-1 and combination treatment (Figure 5F), whereas no noticeable changes occurred following the Foretinib monotherapy. This indicates that the decreased PD-1 levels on T cells following the combination therapy dependent upon αPD-1. This observation indicated that the antitumor immune response was enhanced by the combination treatment.

The infiltration of TAMs in the TME was also assessed after treatment. The combination treatment reduced the percentage of TAMs (Figure 5G) and promoted M2 phenotype polarization toward the M1 phenotype (Figure 5H and Supplementary Figure 6). Further study of functional genes via qPCR suggested that the combination therapy increased the expression of the proinflammatory genes such as NOS2 and TNF-α (Figure 5I), which represented the M1 phenotype TAMs and exert antitumor activity; whereas the expression of M2 phenotype TAM genes such as CD206 and Arg1 were decreased (Figure 5J). Following the combination therapy, the CT26 tumors displayed the same lack of MDSC changes as in the MC38 tumor (Supplementary Figure 4B).

Moreover, the levels of PD-L1 in the CT26 tumor tissues were determined by western blotting after Foretinib treatment, which demonstrated that PD-L1, p-JAK2 and p-STAT1 (included S727 and Y701) were significantly increased (Supplementary Figure 7), which was similar to the MC38 tumor (Figures 1J,K). This result means that it is likely that Foretinib caused increased PD-L1 levels via the activation of the JAK2-STAT1 pathway in the CT26 tumors.

### Combined Treatment Enhanced the Peripheral Immune Profile for Both Models

Since the peripheral immune plays a positive role in antitumor therapies, relevant immune cells in peripheral blood and spleen were detected by FCM. The results suggested that T cells were increased and MDSCs were decreased in the peripheral blood and spleen for both models (Supplementary Figure 8). Tregs were dramatically decreased in the spleen in the MC38 model after combination treatment (Supplementary Figure 8B). Additionally, macrophage was markedly decreased in the spleen for both models and in the peripheral blood of the CT26 model (while they remained unchanged in peripheral blood of the MC38 model) (Supplementary Figure 8). These results suggested that the combination therapy enhanced the peripheral immune profile *in vivo*.

### Combined Treatment Inhibited Tumor Metastasis of CT26-Luc Colon Cancer to the Lung

**FIGURE 6:**
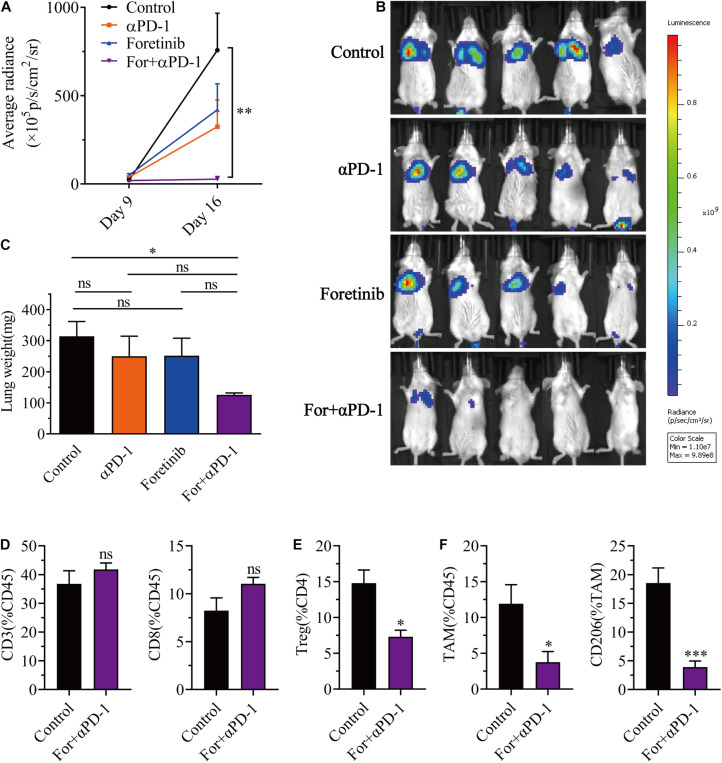
Combination therapy inhibited CT26-Luc tumor metastasis to the lung. CT26-Luc cells (2 × 10^5^) were intravenous injected into the BALB/c mice. On the third day after transplantation, the mice were randomly allocated to either the control or treatment groups. Drugs were given as described in “Materials and Methods” section. **(A,B)** The individual tumor load was evaluated via bioluminescence nine and 16 days after inoculation [**(A)**, the changes of bioluminescence in each group; **(B)**, bioluminescence images on day 16] (*n* = 5). **(C–F)** After treatment, the lungs from the mice in each group were weighed **(C)** and the CD3^+^, CD8^+^, and CD4^+^ T cells **(D)**, Tregs **(E)**, TAMs were detected, plus the expression of CD206 in the TAMs **(F)** were identified using FCM (*n* = 5). The data is presented using the mean ± SEM where applicable, * (*P* ≤ 0.05), ** (*P* ≤ 0.01), *** (*P* < 0.001), **** (*P* < 0.0001), and ns (no statistical significance, *P* ≥ 0.05).

In a similar manner to previous research (Qian et al., 2009), Foretinib inhibited the metastasis of CT26-Luc colon cancer cells (Supplementary Figure 9). To further determine the applicability of the combination therapy to advanced CRC, the effects upon colon cancer metastasis were assessed using a colon cancer lung metastasis model (CT26-Luc). The results demonstrated that both αPD-1 and Foretinib could inhibit cancer metastasis to the lung to some extent (Figure 6A) following injections of CT26-Luc colon cancer cells into mice. However, the inhibition was remarkably enhanced following the combination therapy, for which the bioluminescence was the lowest (Figures 6A,B). After treatment, the weight of lungs in each group were compared, the weight of lungs in the combination treatment was lower than other treatments and the control (Figure 6C). To study the effect of the combination therapy on the TME in the lungs, the T cells and TAMs were assessed after treatment. It was demonstrated that the T cells were increased slightly (Figure 6D), whereas the Tregs, TAMs and the M2 phenotype TAMs were decreased significantly after combination therapy (Figures 6E,F), that means the suppressive TME in lung was ameliorated, and the antitumor immune response was promoted.

Taken together, this data provided evidence that the combination treatment reduced Tregs, TAMs, inhibited TAM polarization toward M2 phenotype, and triggered a successful antitumor metastasis immune response.

## Discussion

Breakthroughs related to cancer immunotherapies have become a promising approach to cure cancer based upon the success of ICB (Couzin-Frankel, 2013; Baumeister et al., 2016; Kaiser and Couzin-Frankel, 2018). The response rates of this therapy are higher than the traditional therapy protocols, but not all types of cancer can benefit from ICB, only 20–40% of cancer patients have a favorable response, and CRC is at the lower end of the spectrum (Fritz and Lenardo, 2019). To enhance the antitumor effect, various trials have sought to establish combination treatments that utilize ICIs and other therapies, such as chemotherapy, targeted therapies, radiotherapy, and other immunotherapies (Postow et al., 2015; Hahn et al., 2017). In this study, Foretinib was combined with anti-PD-1 antibody, which significantly enhanced the antitumor effect.

Many studies have reported that RTKs inhibitors such as cabozantinib (Lu et al., 2017; Patnaik et al., 2017), lenvatinib (Kimura et al., 2018; Gunda et al., 2019), and sorafenib (Cabrera et al., 2013; Chen et al., 2014, 2015) not only inhibited angiogenesis via inhibiting the VEGF/VEGFR2 pathway and ameliorated the TME, but also improved the outcomes for ICI therapeutics. Likewise, the study contained herein identified that Foretinib inhibits the expression of VEGFR2 and inhibited angiogenesis in MC38 tumor tissues. Foretinib also ameliorated the suppressive TME and peripheral immune profile by increasing the infiltration of T cells and decreasing the number of CD11b positive myeloid cells. It is noteworthy that CD11b positive myeloid cells are previously been shown to be comprised of various cellular subtypes, most of them are immunosuppressive cells, such as TAMs and MDSCs (Engblom et al., 2016; Nguyen et al., 2018).

Herein, Foretinib was shown to increase the expression of PD-L1 via activating the JAK2-STAT1 pathway. However, Foretinib also activated other pathways in MC38 cells, such as the AKT/mTOR and MAPK pathways (data not shown), which have been associated with the expression of PD-L1, however, these pathways were inhibited in other tumor cells, such as human colon cancer cell line KM12SM, human esophageal adenocarcinoma cell line OE33, human gastric cancer cell line MKN45 and human lung carcinoma cell lines NCI-H1993 (Liu et al., 2011; Goltsov et al., 2018; Nishiyama et al., 2018; Sohn et al., 2020). Furthermore, Foretinib enhanced the phosphorylation of STAT3 in MC38 cells (data not shown), which contributes to and promotes immune suppression (Yu et al., 2009). These results suggested that Foretinib may regulate the state of cancer cells via several pathways in different cells and future research on the complex regulatory network might be warranted.

It has been proposed that increased expression of PD-L1 could enhance the antitumor effects of the anti-PD-1 antibody for many drugs (Chiang et al., 2019; Deng et al., 2019; Fournel et al., 2019; Huang et al., 2020). Additionally, JAK2-STAT1 pathway activation, especially increase STAT1 phosphorylation at Y701 has been proposed to be a potential biomarker for anticancer immunotherapy within the tumor (Koromilas and Sexl, 2013; Nakayama et al., 2019).

This study demonstrated that monotherapies utilizing Foretinib inhibited tumor growth, but following the cessation of treatment the tumors progressed rapidly. The combination of Foretinib with anti-PD-1 antibody significantly enhanced the antineoplastic function and prolonged the survival of mice bearing MC38 and CT26 tumors. This effect was more prominent for the CT26 tumors, for which nearly an 80% rate of tumor regression without recurrence was observed over 120 days; whereas a 50% rate was observed for the MC38 tumor. This phenomenon may be because CT26 cells form highly immunogenic tumors, which have been shown to have a better response to ICB than MC38 tumors (Mosely et al., 2017; Kristensen et al., 2019).

It is widely recognized that high T cell infiltration into tumors has been associated with a high likelihood of a better prognosis and could be used as a biomarker to predict the effects of anti-PD-1 antibody therapies (Huang et al., 2017). Herein, T cell infiltration was rarely observed in both the tumor models, however, the infiltration was improved to the greatest extent after the administration of the combination treatment. It has been reported that Foretinib promote apoptosis, induce programmed cell death and inhibit angiogenesis by targeted multiple tyrosine kinases, these could release antigens and jumpstart the so-called cancer-immunity cycle, secrete chemoattractant to enhancing T cells infiltration (Huynh et al., 2012; Chen et al., 2017; Kogata et al., 2018; Liu et al., 2019; Hack et al., 2020; Petroni et al., 2021). And in our study, the genes associated with T cell recruitment were enriched within the tumor after the combination treatment, this implies there were more T cells infiltration in tumor. Furthermore, the combination treatment markedly inhibited the expression of PD-1 on T-cells and thereby reversed their functional depletion in the CT26 tumor model. This inhibition was dependent upon the anti-PD-1 antibody, but not Foretinib.

Inhibition of the immunosuppressive functions of Tregs within tumor or a reduction of their numbers have been reported as a method enhance anti-tumor therapies (Zou, 2006). In this study, the combined treatment significantly decreased the infiltration of Tregs for the CT26 tumor, but there were no noticeable changes for MC38 tumor. This may explain why the efficacy of the combination treatment is higher in CT26 tumor than the MC38 tumor. Additionally, these results suggest that different tumor models have different active immune regulatory mechanisms. Indeed, this phenomenon is consistent with previous studies that experienced inconsistent change trends across different models for other RTKs, such as Sorafenib (Chen et al., 2014, 2015).

It is clear that TAMs play a pivotal role in tumor progression and resistance to the effects of PD-1 blockade (Biswas and Mantovani, 2010; Lewis et al., 2016; Mantovani and Locati, 2016), which is closely associated with a poor prognosis (Asgharzadeh et al., 2012; Koromilas and Sexl, 2013; Hambardzumyan et al., 2016). In this study, TAMs were the primary leukocyte infiltrates for both of the tumor models. More than 50% of the TAMs expressed CD206, which was identified as the marker of the M2 phenotype TAMs and were associated with immunosuppressive effects in tumors. Compared to the monotherapies, the Foretinib and anti-PD-1 antibody combination therapy exhibited a more effective rate of TAM reduction and a greater inhibition of the polarization of TAMs toward the M2 phenotype. Indicating that the TME was ameliorated in the tumors of mice receiving the combination treatment. Whilst the M2 phenotype TAMs were decreased for both of the models, the TAMs were decreased to a lesser extent in the CT26 tumor when compared to the significant decrease in the MC38 tumors after Foretinib treatment.

Since the peripheral immune profile can play a positive role in achieving greater antitumor effects, T-cells, Tregs, macrophages and MDSCs in the peripheral blood and spleen of both tumor models were analyzed using FCM following the different treatments. These results showed that the combination treatment increased the percentage of T-cells, especially CD8^+^ T cells, and decreased the percentage of MDSCs in the peripheral blood and spleen for both tumor models. This indicates that the anti-tumor functions of the peripheral immune were enhanced. However, the number of Tregs were decreased in the spleens of the MC38 model and unchanged in the CT26 model after combination treatment, whereas the tumor tissues were observed to have the opposite trend. Macrophages were found to diminished in the blood of the CT26 model, but unchanged in the MC38 model. These results indicated that there are complex immune regulatory mechanisms occuring *in vivo*, many of which could impact this combination therapy and might need to be further explored in future studies.

The combination treatment also effectively reduced the metastasis of CT26-Luc cells to the lungs when compared with the monotherapies. Furthermore, the number of T cells were increased moderately and there was a decrease in the number of Tregs, TAMs and M2 phenotype TAMs in the lungs after the combination therapy, which indicates that there was an amelioration of the immune microenvironment in the lungs. The effect of Foretinib for the inhibition of CT26-Luc cell metastasis *in vitro* was pronounced in this study. However, the inhibitory effects of metastasis to the lung *in vivo* were poor. This indicates that there may be additional relevant immune regulatory mechanisms in TME in the lung, which could be worth further exploration.

## Conclusion

In summary, this study describes that Foretinib (an RTKs inhibitor) therapy increases PD-L1 levels via the activation of the JAK2-STAT1 pathway. This in turn increased the therapeutic response to the anti-PD-1 antibody through the reprogramming of the immunosuppressive TME. This included enhanced T cell infiltration and function, inhibited TAMs and reversed TAM polarization toward the M2 phenotype. Moreover, Foretinib enhanced the peripheral immune profiles in the murine tumor models. This investigation forms a rational basis for the further exploration of a Foretinib/anti-PD-1 combination treatment strategy to enhance immunotherapy outcomes for CRC patients.

## Materials and Methods

### Chemicals and Reagents

Foretinib was purchased from Lollane Biological Technology (Shanghai, China), and the anti-mouse PD-1 Ab (CD279) (clone RMP1-14) was purchased from BioXCell (West Lebanon, United States). DMSO was supplied by Solarbio Science and Technology (Beijing, China). Red Blood Cell Lysis Buffer (ACK Lysis Buffer, Beyotime, China) was used to lyse erythrocytes. Monoclonal antibodies against CD3 (11014), CD8 (11068), and CD31 (11063-2) were used for immunofluorescence, which were purchased from Servicebio (Wuhan, China). Monoclonal antibodies against CD16/32 (553142), CD45 (553079), CD3 (557596), CD8 (553079), CD4 (560782), IFN-γ (554412), CD25 (557192), Foxp3 (563101), Ly-6G/Ly-6C (Gr-1) (553128) and CD11b (557657), and Leukocyte Activation Cocktail with BD GolgiPlug (550583) for FCM were purchased from BD Biosciences (San Jose, United States). Monoclonal antibodies against CD4 (100559), PD-1 (135220), PD-L1 (124331), F4/80 (123110) and MHC class II (107625) for FCM were purchased from BioLegend (San Diego, United States). Monoclonal antibodies against CD206 (17-2061-82) for FCM was purchased from eBioscience (Worcester, MA, United States). A Transcription Factor Buffer Set (562725) kit for intracellular Foxp3^+^ TReg staining was purchased from BD Biosciences (San Jose, United States). Mononuclear cells in mouse spleens were extracted using Histopaque-1083 (Sigma-Aldrich, United States). Fixation Buffer (420801) and Intracellular Staining Permeabilization Wash Buffer (10×) (421001) kit for intracellular IFN-γ staining were acquired from BioLegend (San Diego, United States). Antibodies against VEGFR2 (2479S) and phospho-STAT1 (Y701) (9167) for western-blot was purchased from Cell Signaling Technology (Danvers, MA, United States), Abs of phospho-JAK2 (ET1607-34), JAK2 (ET1607-35), PD-L1 (ET1701-41), STAT1 (ET1606-39), phospho-STAT1 (S727) (ET1611-20) for western-blot were purchased from HUABIO (Hangzhou, China). A monoclonal antibody against β-actin (TA-09), Horseradish peroxidase (HRP)-conjugated goat anti-rabbit IgG (ZB-2301) and HRP-conjugated goat anti-mouse IgG (ZB-2305) for western-blotting were purchased from ZSGB-BIO (Beijing, China).

### Mice and Cell Lines

Six to eight weeks-old C57BL/6 and BALB/c female mice were purchased from Beijing Vital River Laboratory Animal Technology Co., Ltd. (Beijing, China). The animals were housed and maintained under optimal conditions of light, temperature, and humidity with free access to food and water.

MC38, CT26 and CT26-Luc (a CT26 cell line that expresses luciferase) mouse colon carcinoma cell lines, and HT29, HCT116, SW480 human colon cancer cell lines were obtained from the Type Culture Collection of the Chinese Academy of Sciences (Shanghai, China). Cells were cultivated in DMEM or RPMI 1640 (Hyclone, United States) containing 10% fetal bovine serum (Bioind, IL), 100 U/ml penicillin (Thermo Scientific, United States), and 100 mg/ml streptomycin (Thermo Scientific, United States) and were cultured in a humidified 5% CO_2_ atmosphere at 37°C in incubator.

### Animal Experiments

For the subcutaneous tumor model, a total of 1 × 10^6^ MC38 cells or 5 × 10^5^ CT26 cells were resuspended in 100 μL serum-free medium and injected subcutaneously into the right flank of the C57BL/6 or BALB/c mice. Tumor sizes were measured with a digital caliper every 3 days. Tumor volume (mm^3^) was estimated using the following formula: tumor volume = (long axis) × (short axis)^2^ × 0.5. Mice were sacrificed when the tumor volume reached ∼2,000 mm^3^ and the survival of the mice was recorded daily.

For the generation of the lung metastasis tumor model, 2 × 10^5^ CT26-Luc cells in 100 μL serum-free medium were injected intravenously into BALB/c mice via the tail vein, and the development of tumors in the lungs was monitored using IVIS Lumina III (PerkinElmer) after intraperitoneal injection of D-luciferin potassium (meilunbio, China).

Foretinib (5 mg/kg) treatment was initiated via oral gavage every day on the sixth day after tumor cell inoculation for the subcutaneous tumor model and the second day for the lung metastasis tumor model. The αPD-1 monoclonal antibody was administered by intraperitoneal injection every 3 days for a total of four injections. The tumor-bearing mice were anesthetized on the indicated days and tissues were harvested for further analysis and measurement.

All the animal studies were approved by Sichuan University’s Institutional Animal Care and Use Committee and were performed in accordance with the institutional guidelines.

### CD8^+^ T Cell Depletion

For in vivo depletion of CD8 T cells, neutralizing antibodies of αCD8 (YTS169.4 Clone, 250 μg/mouse) were intraperitoneally injected on day 2, 5, 8, 11, 14, 17 after MC38 cells inoculation and the tumor growth was evaluated.

### FCM Analysis

Tumors and spleens from the subcutaneous models and lungs from the lung metastasis models were harvested after treatment and were digested at 37°C for 1 h in RIPM-1640 or DMEM medium containing 1 mg/mL collagenase type IV (Roche, CH) and 0.5 mg/ml DNase I (Roche, CH). Then passed through a 70-mm cell strainer and washed twice with PBS containing 1% BSA. After the erythrocytes were lysed, the single cells were blocked with a CD16/32 blocker and were stained for 30 min at 4°C using the following anti-mouse antibodies: CD45, CD3, CD4, CD8, CD25, PD-1, F4/80, CD11b, CD206, MHC-II, and Gr-1.

For the intracellular IFN-γ staining, spleens were harvested and the mononuclear cells were extracted and incubated in 24-well, flat-bottom plates with Leukocyte Activation Cocktail with BD GolgiPlug for 4 h at 37°C in a humidified 5% CO_2_ incubator and subsequently stained with antibodies against CD45, CD3, CD4, and CD8 for 30 min at 4°C. The cells were then fixed and permeabilized with Fixation Buffer and Intracellular Staining Permeabilization Wash Buffer according to the kits’ protocol and then stained with an anti-IFN-γ antibody.

For the Foxp3^+^ Tregs staining, the surface markers were stained, fixed and permeabilized with a Transcription Factor Buffer Set according to the kit’s protocol, then stained with the anti-Foxp3 antibody. To identify live cells, Fixable Viability Stain 450 (BD Biosciences, 562247) was used. Data was acquired on LSR Fortessa flow cytometer and analyzed using Flow Jo V10.0 software (BD Biosciences, United States).

### Real-Time PCR

Total RNA was collected from tumor tissues using an Animal Total RNA Isolation Kit (Foregene Co., Ltd, China) following the manufacturer’s protocol. cDNA was prepared using HiScript II Q RT SuperMix for qPCR (+gDNA Wiper) (Vazyme Biotech Co., Ltd., China). The qPCR was performed with AceQ SYBR qPCR Master Mix (Vazyme Biotech Co., Ltd., China) and the gene expression was normalized to the housekeeping gene ActB.

### Western-Blot

Cells were treated with varying concentrations of Foretinib for 24 h and washed twice with PBS buffer and the tumor tissues were ground with liquid nitrogen following the drug treatment protocol, prior to lysis with RIPA buffer (Beyotime, China) containing protease inhibitor cocktail (MCE, United States) and phosphatase Inhibitor Cocktail I (MCE, HY-K0021), respectively. After sonication, the supernatant was obtained by centrifugation at 4°C and the protein concentrations were determined using a BCA protein assay kit (Thermo Scientific, United States). Total protein (50 or 100 μg) was separated by SDS-PAGE and then transferred onto a PVDF membrane (Millipore, IPVH00010). After blocking with 5% skimmed milk, the membranes were incubated with the appropriate antibody concentration (1:500-1:1000): JAK2, Phospho-JAK2, PD-L1, STAT1, phospho-STAT1 (including S727 and Y701), VEGFR2 and β-actin overnight at 4°C. HRP-conjugated goat anti-rabbit IgG and HRP-conjugated goat anti-mouse IgG were used as the secondary antibodies. The FUSION FX. EDGE System (VILBER LOURMAT) was used for the imaging and protein levels were based upon the signal intensity. The protein expression was normalized to β-actin.

### Cytokine Assay

Peripheral blood was collected from the mice and the IFN-γ and TNF-α cytokine levels were evaluated in the plasma following separated by centrifugation at 4°C and then stored at –80°C. The concentration of IFN-γ and TNF-α in the plasma were assessed using a QuantiCyto Mouse IFN-γ ELISA kit (NeoBioscience, China) and a QuantiCyto Mouse TNF-α ELISA kit (NeoBioscience, China) according to the manufacturer’s protocol.

### Immunohistochemistry

For the immunohistochemistry (IHC) analysis, formalin-fixed paraffin-embedded tissue sections were deparaffinized and the slides were stained following antigen retrieval in EDTA buffer. After washing in PBS, the slides were incubated with 10% BSA for 30 min, Rat anti-CD3 (1:200) was added to the slides and incubated overnight at 4°C. After washing in PBS, goat-anti-rat FITC conjugated secondary antibodies were applied and incubated for 50 min at room temperature prior to being washed with PBS. This process was also used for the mouse anti-CD8 antibody or Rat anti-CD31 (1:200) antibody with the cy3-conjugated goat-anti-rat antibody. After PBS washes, DAPI was added and the slides were incubated for 10 min at room temperature. The slides were then rinsed with PBS and water before being imaged by confocal fluorescence microscopy (ZEISS, LSM 880, DE).

### Statistical Analysis

The results were presented using the mean plus/minus the standard error of the mean (±SEM) where appropriate. All data analysis was performed using GraphPad Prism software (version 8.0). A One-way ANOVA and unpaired *t*-test was used for the statistical analyses of the data where appropriate. Animal survival was presented using Kaplan–Meier survival curves and analyzed via the log-rank test. A value of *P* ≤ 0.05 was used as the threshold to reject the null hypothesis and determine the statistical significance of the data. The following standard form of abbreviation was used to indicate the significance of the data displayed in the figures: ^∗^ (*P* ≤ 0.05), ^∗∗^ (*P* ≤ 0.01), ^∗∗∗^ (*P* ≤ 0.001), ^****^ (*P* ≤ 0.0001) and ns (no statistical significance, *P* ≤ 0.05).

## Data Availability Statement

The original contributions presented in the study are included in the article/Supplementary Material, further inquiries can be directed to the corresponding author/s.

## Ethics Statement

The animal study was reviewed and approved by Ethics Committee of the State Key Laboratory of Biotherapy, Sichuan University, China.

## Author Contributions

YF and YP contributed equally to this work. YF, YP, and JY conceived and designed the experiments. YF, YP, and JM were involved in the acquisition and analysis of the data. YF, YP, and XJ performed the animal studies. YF, MW, and YiL were involved in the qPCR assay. YF, SZ, JM, LZ, CY, and CH conducted FCM and IHC assay. YaW, YuY, YuW, and LG provided advice. YF, YaY, TK, and YuL were involved in Western-Blot. YF wrote the manuscript. YF, YP, QL, and JY reviewed and/or revised of the manuscript. LG and JY supervised the study. All the authors contributed to the revision of the manuscript and approved the final version for publication.

## Conflict of Interest

The authors declare that the research was conducted in the absence of any commercial or financial relationships that could be construed as a potential conflict of interest.

## Supplementary Material

Supplementary Figure 1 Foretinib increased level of PD-L1 and activated the JAK2-STAT1 pathway in colon cancer cells. The expression of PD-L1, jak2, stat1, phosphor-stat1 and β-actin was detected by Western-blot in CT26, HCT116, HT29, and SW480 colon cancer cells that was treated with different concentrations of Foretinib for 24 h.

Supplementary Figure 2 Foretinib regulated Peripheral immune microenvironment. The mice were successively gavage with Foretinib at the dose of 5 mg/kg for 15 days when the tumor volume was approximately reached to 100 mm^3^. After the administration completed, the percentages of CD3^+^, CD4^+^ and CD8^+^ T cells in the peripheral blood **(A)** and spleen **(B)** were detected by flow cytometry (*n* = 6 mice per group). The data is presented using the mean ± SEM where applicable, ^∗^ (*P* ≤ 0.05), ^∗∗^ (*P* ≤ 0.01), ^∗∗∗^ (*P* < 0.001), ^****^ (*P* < 0.0001), and ns (no statistical significance, *P*≥0.05).

Supplementary Figure 3 Combination therapy enhanced the function of T cell in MC38 model. After treatment, the spleen cells were cultured 4 h with Leuko Act Cktl With GolgiPlug, and the IFN-γ^+^ T cells were detected by flow cytometry (*n* = 5 mice per group). **(A)** Left, flow-cytometric analysis of IFN-γ^+^ CD4^+^ T cells. Right, the percentage of IFN-γ^+^ T cells in CD4^+^ T cells. **(B)** Left, flow-cytometric analysis of IFN-γ^+^ CD8^+^ T cells. Right, the percentage of IFN-γ^+^ T cells in CD8^+^ T cells. **(C–E)** The percentages of PD-1^+^ cells in CD3^+^, CD4^+^, CD8^+^ T cells were analysed by FCM (*n* = 5 mice per group). **(F,G)** The cytokines of IFN-γ and TNF-α were detected in plasma by ELISA (*n* = 5 mice per group) when finished treatment in MC38 tumor. The data is presented using the mean ± SEM where applicable, ^∗^ (*P* ≤ 0.05), ^∗∗^ (*P* ≤ 0.01), ^∗∗∗^ (*P* < 0.001), ^****^ (*P* < 0.0001), and ns (no statistical significance, *P* ≥ 0.05).

Supplementary Figure 4 Combination therapy not effect MDSCs infiltration in both tumors. After the administration completed, the percentages of MDSC cells in MC38 tumor **(A)** and CT26 tumor **(B)** were detected by flow cytometry (*n* = 6 mice per group). The data is presented using the mean ± SEM where applicable, ^∗^ (*P* ≤ 0.05), ^∗∗^ (*P* ≤ 0.01), ^∗∗∗^ (*P* < 0.001), ^****^ (*P* < 0.0001), and ns (no statistical significance, *P* ≥ 0.05).

Supplementary Figure 5 Combination therapy enhanced the expression of T cell recruitment and function related genes and elevated the concentration of cytokines in CT26 model. The expression of T cell recruitment gene **(A)** and T cell function related genes **(B)** were analyzed by qRT-PCR (*n* = 4 mice per group) and the cytokines of IFN-γ **(C)** and TNF-α **(D)** were detected in plasma by ELISA (*n* = 5 mice per group) when finished treatment in CT26 tumor. The data is presented using the mean ± SEM where applicable, ^∗^ (*P* ≤ 0.05), ^∗∗^ (*P* ≤ 0.01), ^∗∗∗^ (*P* < 0.001), ^****^ (*P* < 0.0001), and ns (no statistical significance, *P* ≥ 0.05).

Supplementary Figure 6 Combination therapy inhibited TAMs polarization towards M2-type in CT26 model. The mean fluorescence intensity(MFI) of CD206 [**(A)**, left, the CD206 histogram of flow cytometry. right, percentages of CD206 positive cells] and MHCII **(B)** in TAMs in CT26 tumor were detected by flow cytometry after treatment (*n* = 6 mice per group). The data is presented using the mean ± SEM where applicable, ^∗^ (*P* ≤ 0.05), ^∗∗^ (*P* ≤ 0.01), ^∗∗∗^ (*P* < 0.001), ^****^ (*P* < 0.0001), and ns (no statistical significance, *P* ≥ 0.05).

Supplementary Figure 7 Foretinib enhanced the expression of PD-L1 in CT26 tumor. The level of proteins were detected by Western-blot in CT26 tumor tissue after treatment (left) and the relative protein expression was shown in (right), respectively (*n* = 3 mice per group). The data is presented using the mean ± SEM where applicable, ^∗^ (*P* ≤ 0.05), ^∗∗^ (*P* ≤ 0.01), ^∗∗∗^ (*P* < 0.001), ^****^ (*P* < 0.0001), and ns (no statistical significance, *P* ≥ 0.05).

Supplementary Figure 8 Combination therapy regulated Peripheral immune microenvironment. After the administration completed, the percentages of CD3^+^, CD4^+^, and CD8^+^ T cells, MDSCs, TAMs and Tregs in the peripheral blood and spleen in MC38 tumor model **(A,B)** and CT26 tumor model **(C,D)** were detected by flow cytometry, respectively (*n* = 6 mice per group). The data is presented using the mean ± SEM where applicable, ^∗^ (*P* ≤ 0.05), ^∗∗^ (*P* ≤ 0.01), ^∗∗∗^ (*P* < 0.001), ^****^ (*P* < 0.0001), and ns (no statistical significance, *P* ≥ 0.05).

Supplementary Figure 9 Foretinib inhibited tumor cells invasion and metastasis. The invasion and metastasis of colon cancer cells was detected by Transwell assay after treatment by different concentration Foretinib. Briefly, cells were added into Transwell chamber that already covered matrix glue. Then, added the final concentration were 0.01% DMSO and 1, 2, and 4 μM Foretinib, and added 500 μL RPIM-1640 medium containing 5% FBS to the 24 well plate. Then place the chamber into the plate and cultivated for 24 h. Fixing by methanol, staining by crystal violet and washing two times by dd H_2_O, and taking picture on microscope after drying the chamber. Scale bar, 50 μm.
