# Supplementary material for: Combination Foretinib and Anti-PD-1 Antibody Immunotherapy for Colorectal Carcinoma
Source: Front Cell Dev Biol. 2021 Jul 8;9:689727. doi: 10.3389/fcell.2021.689727 (PMC8298272; doi:10.3389/fcell.2021.689727)
Supplement: Supplementary Figure 1 — Foretinib increased level of PD-L1 and activated the JAK2-STAT1 pathway in colon cancer cells. The expression of PD-L1, jak2, stat1, phosphor-stat1 and β-actin was detected by Western-blot in CT26, HCT116, HT29, and SW480 colon cancer cells that was treated with different concentrations of Foretinib for 24 h. [file Table_1.docx]

Supplementary Material

## Supplementary Figures

##
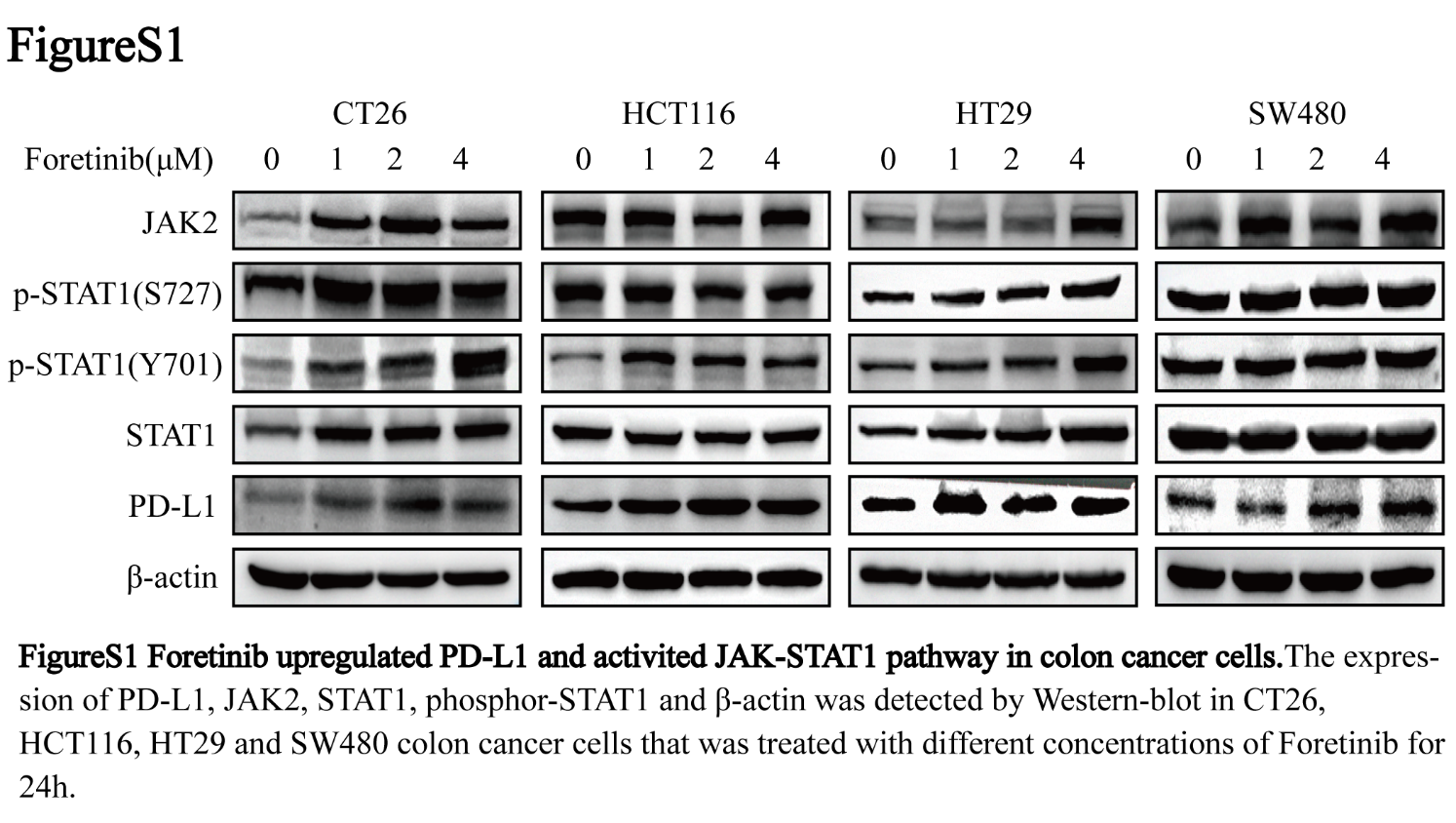


## Supplementary Figure 1 Foretinib increased level of PD-L1 and activated the JAK2-STAT1 pathway in colon cancer cells. The expression of PD-L1, jak2, stat1, phosphor-stat1 and β-actin was detected by Western-blot in CT26, HCT116, HT29 and SW480 colon cancer cells that was treated with different concentrations of Foretinib for 24h.


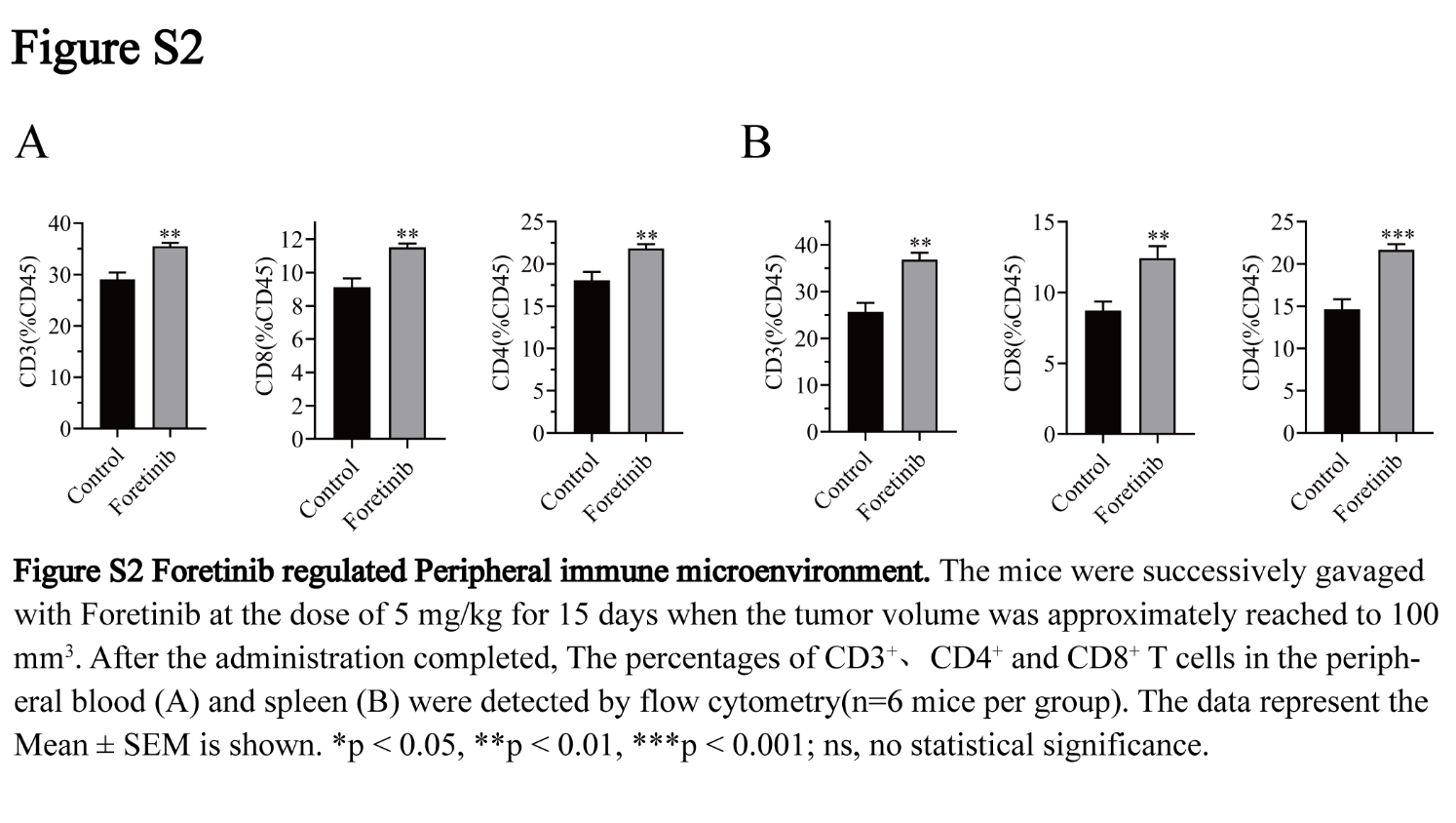


**Supplementary Figure 2 Foretinib regulated Peripheral immune microenvironment.** The mice were successively gavage with Foretinib at the dose of 5 mg/kg for 15 days when the tumor volume was approximately reached to 100 mm^3^. After the administration completed, the percentages of CD3^+^、CD4^+^ and CD8^+^ T cells in the peripheral blood (A) and spleen (B) were detected by flow cytometry(n=6 mice per group). The data is presented using the mean ±SEM where applicable, * (P=< 0.05), ** (P= <0.01), *** (P<0.001), **** (P<0.0001) and ns (no statistical significance, P=>0.05).

**
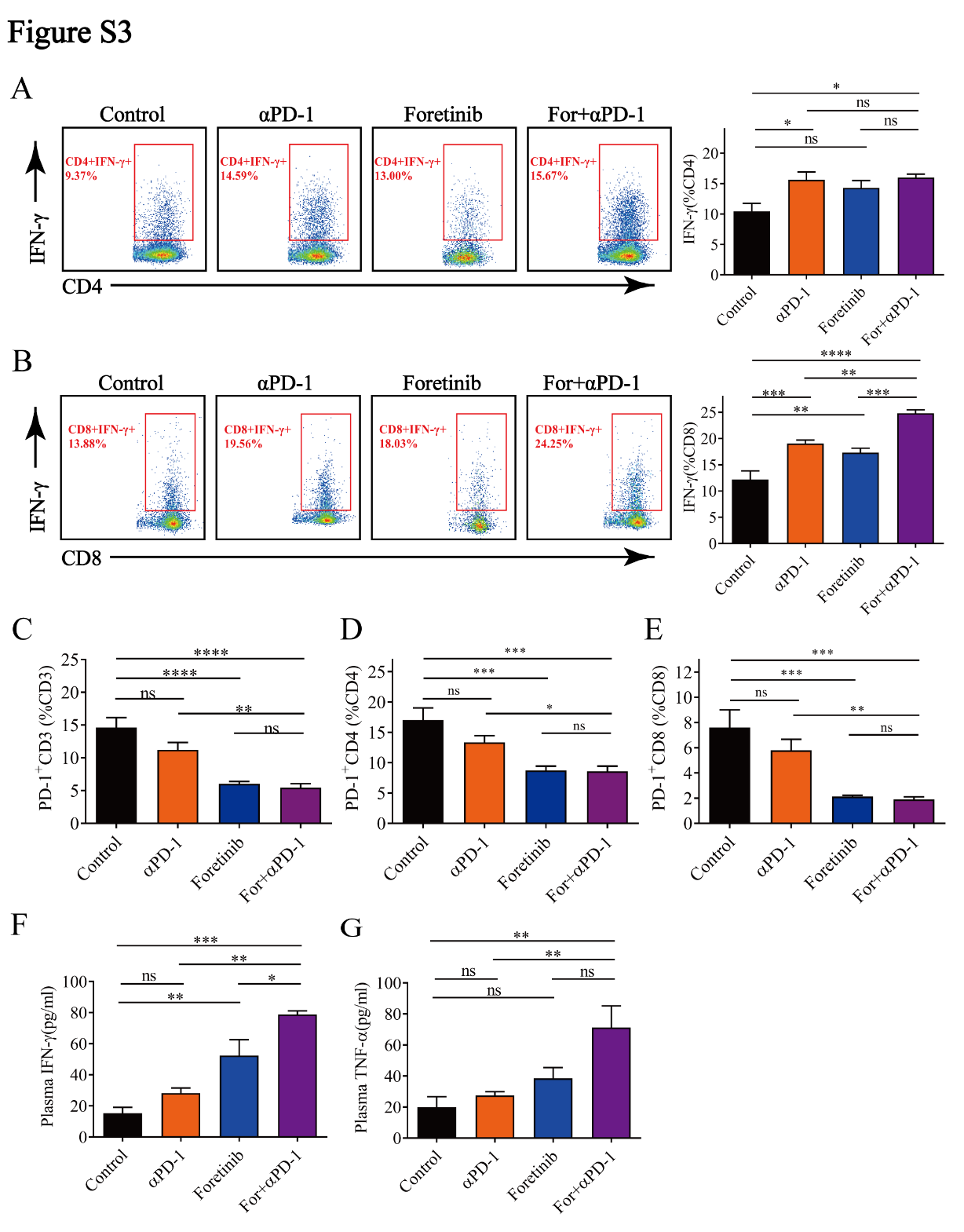
**

**Supplementary Figure3 Combination therapy enhanced the function of T cell in MC38 model.** After treatment, the spleen cells were cultured 4h with Leuko Act CktlWithGolgiPlug, and the IFN-γ^+^ T cells were detected by flow cytometry(n=5 mice per group). (A)Left, flow-cytometric analysis of IFN-γ^+^ CD4^+^ T cells. Right, The percentage of IFN-γ^+^ T cells in CD4^+^ T cells. (B) Left, flow-cytometric analysis of IFN-γ^+^ CD8^+^ T cells. Right, The percentage of IFN-γ^+^ T cells in CD8^+^ T cells. (C-E)The percentages of PD-1^+^ cells in CD3^+^, CD4^+^, CD8^+^T cells were analysed by FCM(n=5 mice per group). (F-G) the cytokines of IFN-γand TNF-αwere detected in plasma by ELISA (n=5 mice per group) when finished treatment in MC38 tumor. The data is presented using the mean ±SEM where applicable, * (P=< 0.05), ** (P= <0.01), *** (P<0.001), **** (P<0.0001) and ns (no statistical significance, P=>0.05).


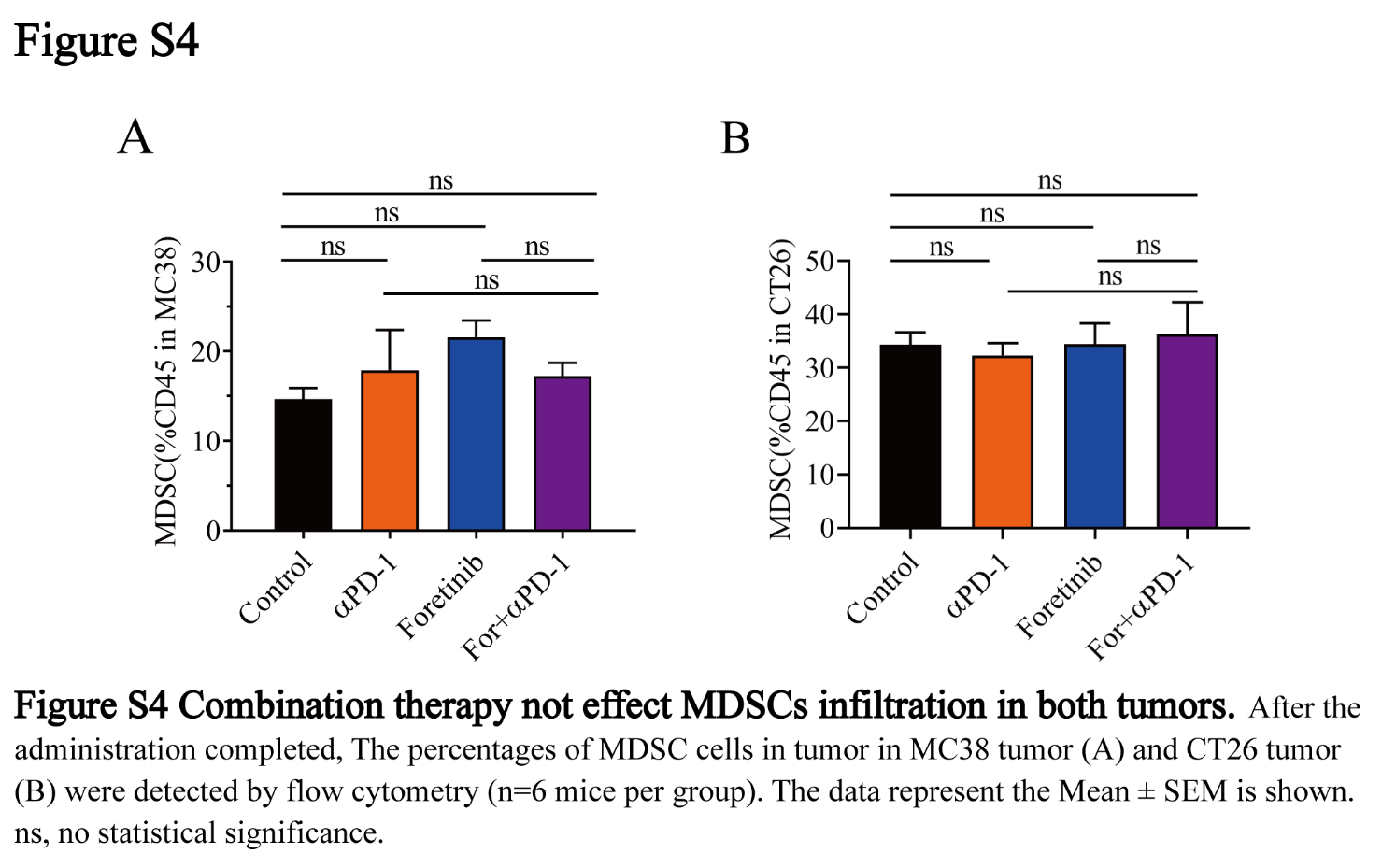


**Supplementary Figure4 Combination therapy not effect MDSCs infiltration in both tumors.** After the administration completed, the percentages of MDSC cells in MC38 tumor (A) and CT26 tumor (B) were detected by flow cytometry (n=6 mice per group). The data is presented using the mean ±SEM where applicable, * (P=< 0.05), ** (P= <0.01), *** (P<0.001), **** (P<0.0001) and ns (no statistical significance, P=>0.05).


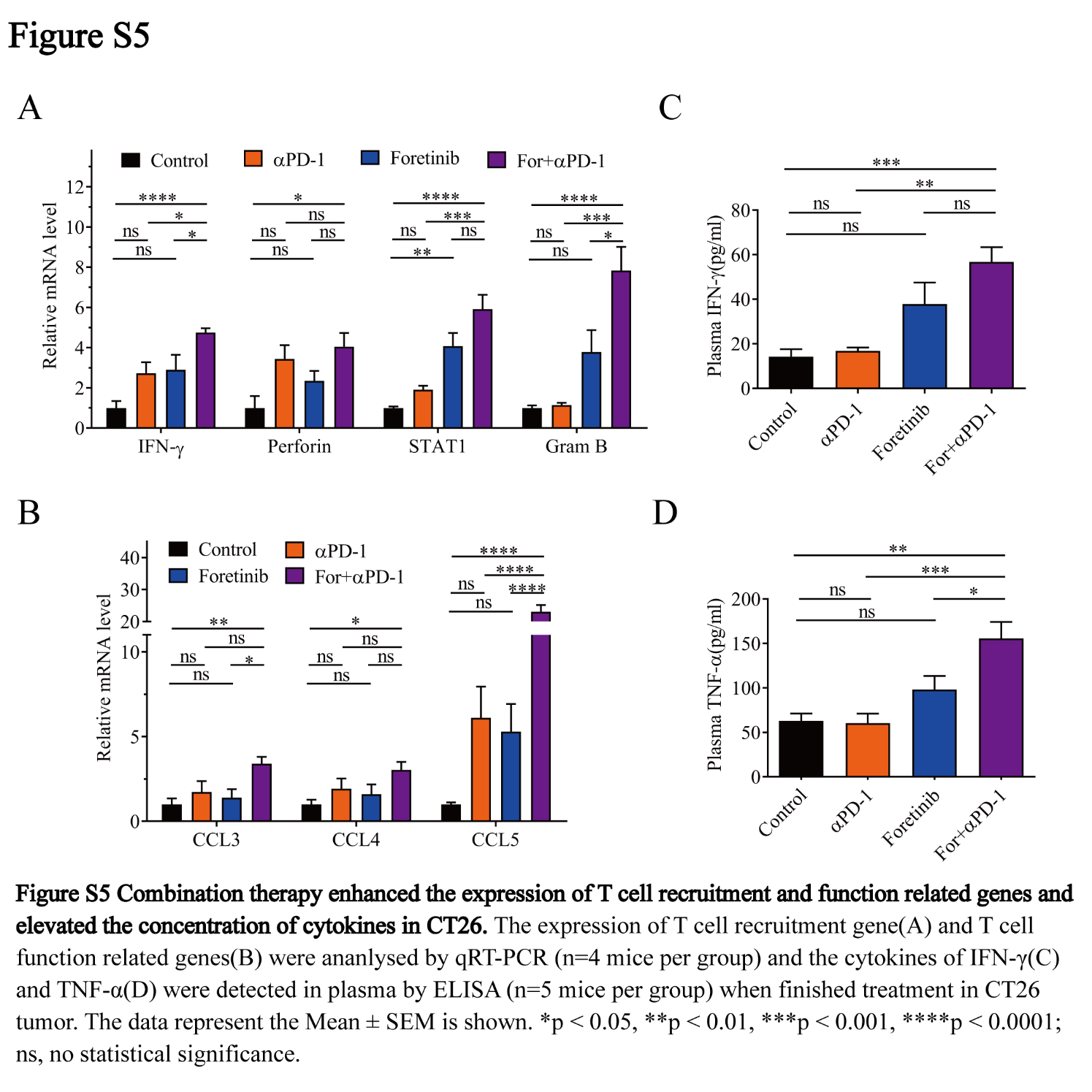


**Supplementary Figure5 Combination therapy enhanced the expression of T cell recruitment and function related genes and elevated the concentration of cytokines in CT26 model.**The expression of T cell recruitment gene(A) and T cell function related genes(B) were analysed by qRT-PCR (n=4 mice per group) and the cytokines of IFN-γ(C) and TNF-α(D) were detected in plasma by ELISA (n=5 mice per group) when finished treatment in CT26 tumor.The data is presented using the mean ±SEM where applicable, * (P=< 0.05), ** (P= <0.01), *** (P<0.001), **** (P<0.0001) and ns (no statistical significance, P=>0.05).


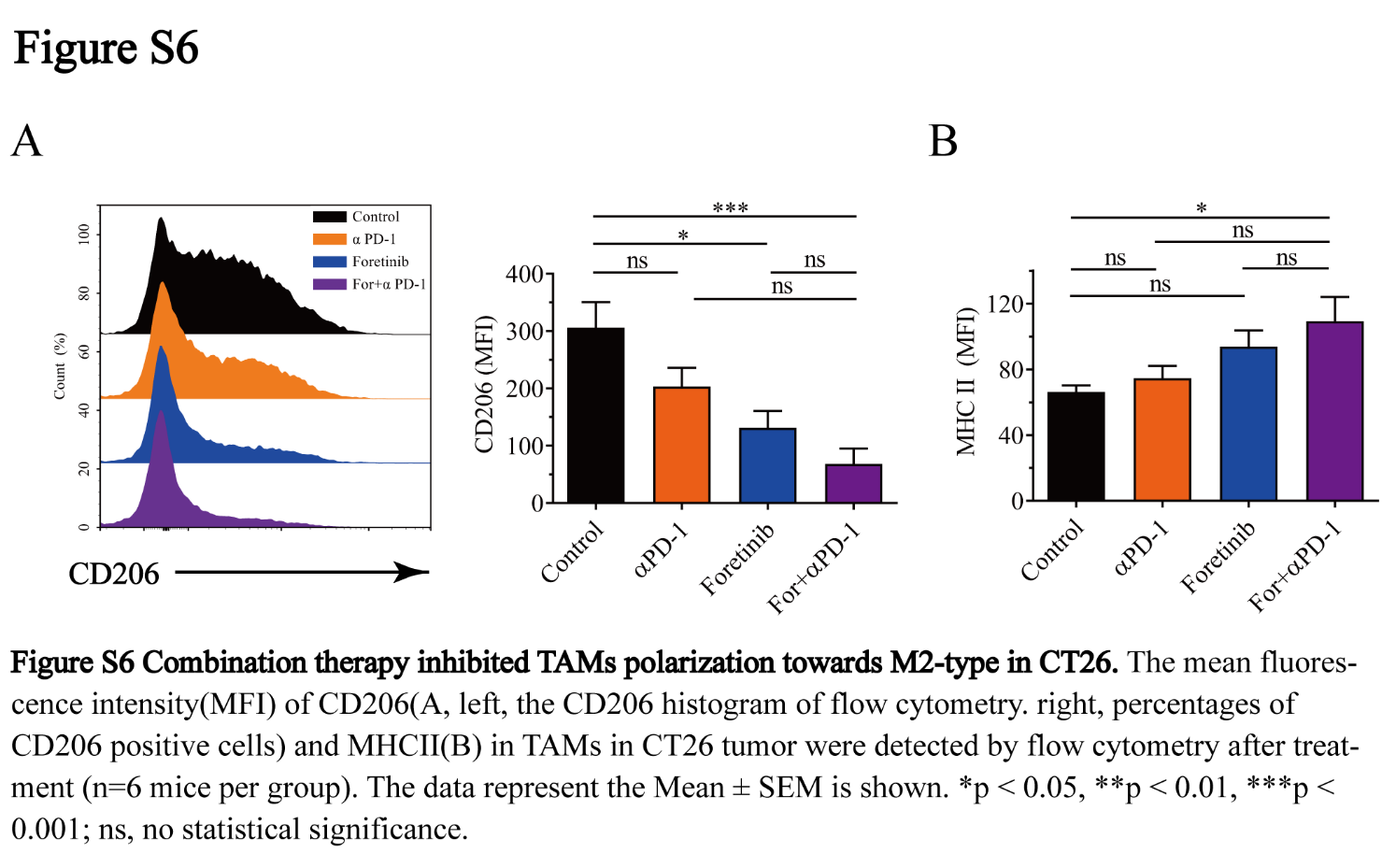


**Supplementary Figure 6 Combination therapy inhibited TAMs polarization towards M2-type in CT26 model.** The mean fluorescence intensity(MFI) of CD206(A, left, the CD206 histogram of flow cytometry. right, percentages of CD206 positive cells) and MHCII(B) in TAMs in CT26 tumor were detected by flow cytometry after treatment (n=6 mice per group). The data is presented using the mean ±SEM where applicable, * (P=< 0.05), ** (P= <0.01), *** (P<0.001), **** (P<0.0001) and ns (no statistical significance, P=>0.05).


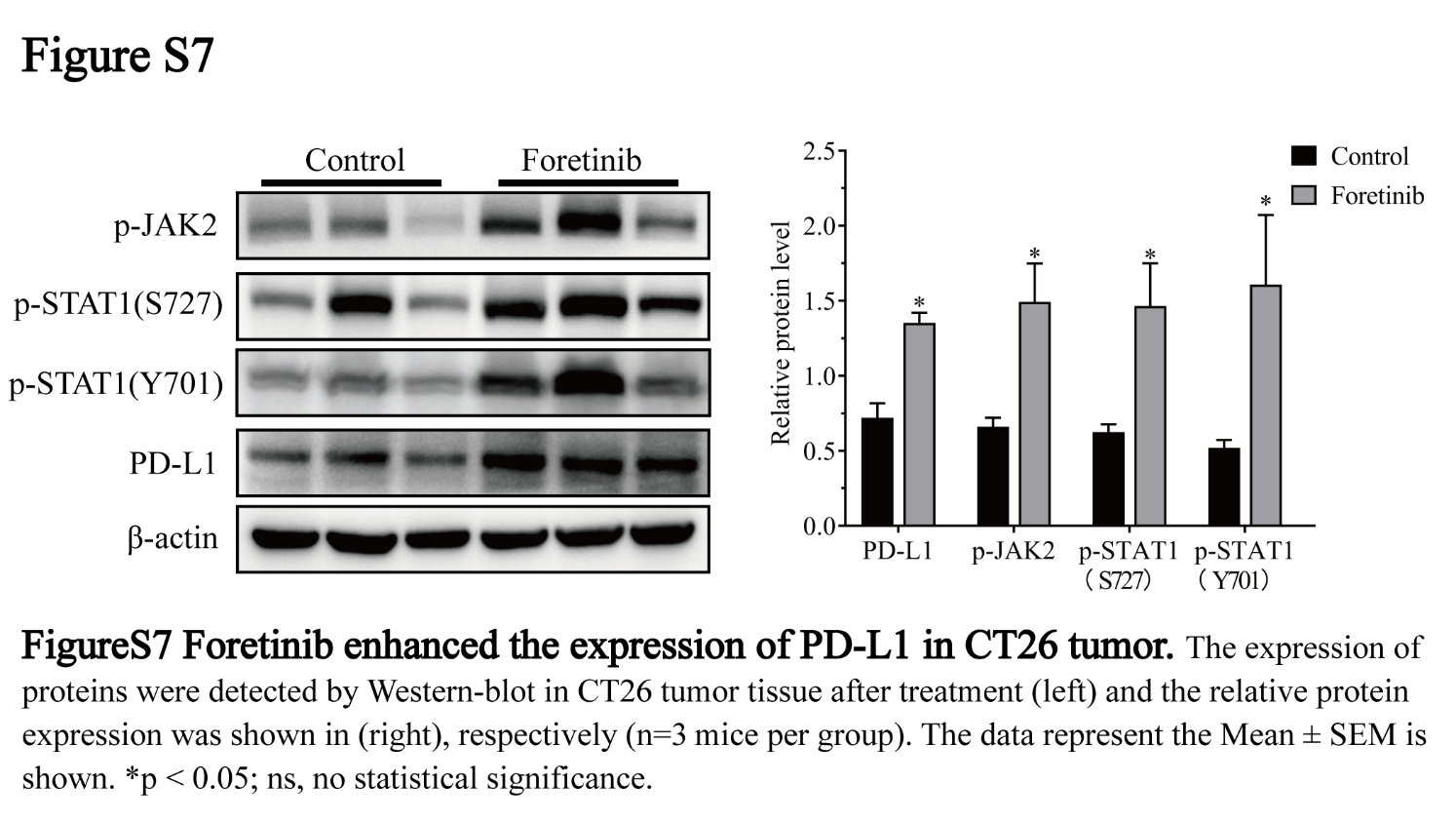


**Supplementary Figure 7 Foretinib enhanced the expression of PD-L1 in CT26 tumor.** The level of proteins were detected by Western-blot in CT26 tumor tissue after treatment (left) and the relative protein expression was shown in (right), respectively (n=3 mice per group). The data is presented using the mean ±SEM where applicable, * (P=< 0.05), ** (P= <0.01), *** (P<0.001), **** (P<0.0001) and ns (no statistical significance, P=>0.05).


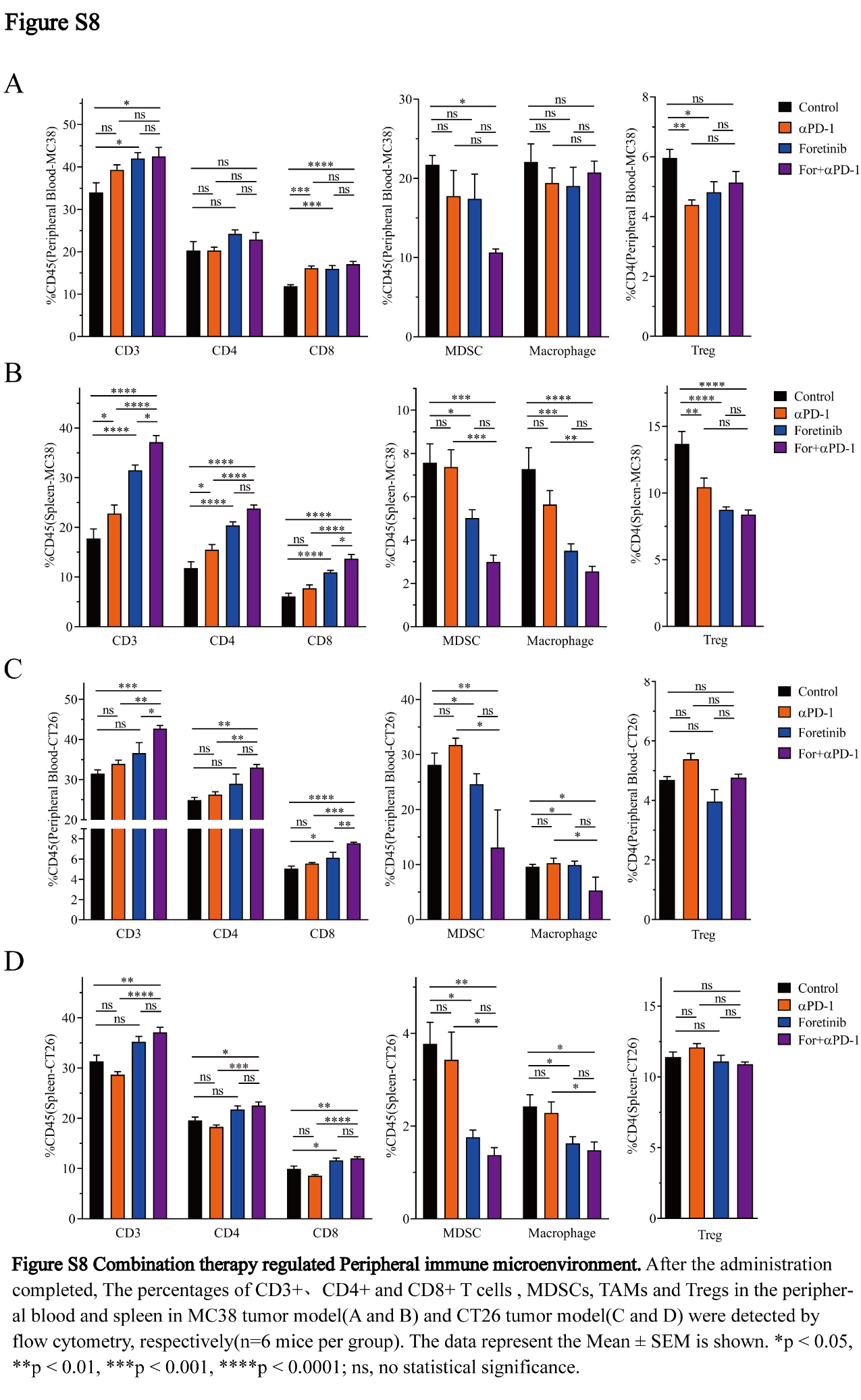
**Supplementary Figure 8 Combination therapy regulated Peripheral immune microenvironment.** After the administration completed, the percentages of CD3^+^、CD4^+^ and CD8^+^ T cells , MDSCs, TAMs and Tregs in the peripheral blood and spleen in MC38 tumor model(A and B) and CT26 tumor model(C and D) were detected by flow cytometry, respectively(n=6 mice per group). The data is presented using the mean ±SEM where applicable, * (P=< 0.05), ** (P= <0.01), *** (P<0.001), **** (P<0.0001) and ns (no statistical significance, P=>0.05).

**
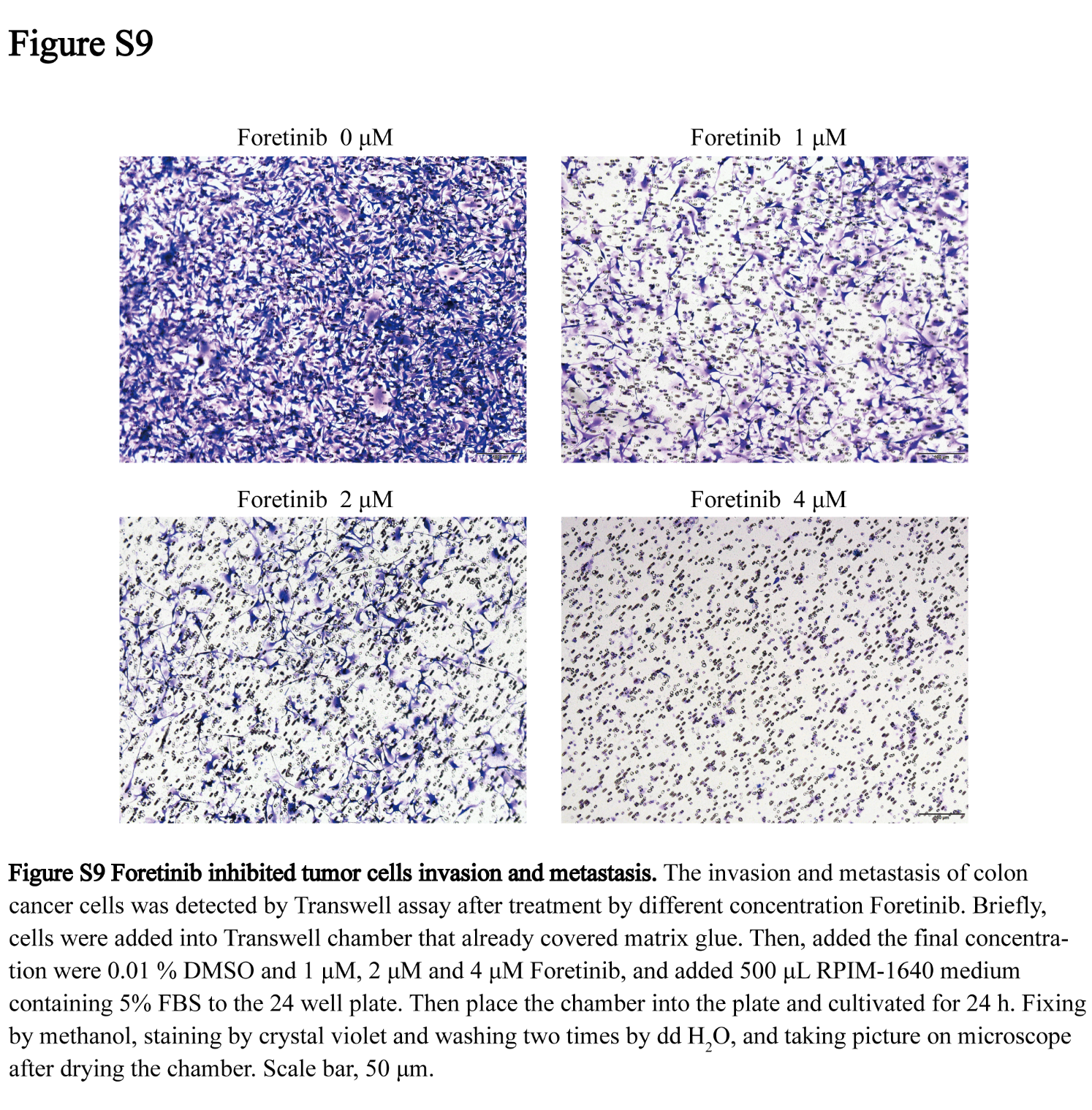
**

**Supplementary Figure9Foretinib inhibited tumor cells invasion and metastasis.** The invasion and metastasis of colon cancer cells was detected by Transwell assay after treatment by different concentration Foretinib. Briefly, cells were added into Transwell chamber that already covered matrix glue. Then, added the final concentration were 0.01 % DMSO and 1 μM, 2 μM and 4 μM Foretinib, and added 500 μL RPIM-1640 medium containing 5% FBS to the 24 well plate. Then place the chamber into the plate and cultivated for 24 h. Fixing by methanol, staining by crystal violet and washing two times by dd H_2_O, and taking picture on microscope after drying the chamber. Scale bar, 50 μm.
